# Egg Intake in Chronic Kidney Disease

**DOI:** 10.3390/nu10121945

**Published:** 2018-12-07

**Authors:** Dina A. Tallman, Sharmela Sahathevan, Tilakavati Karupaiah, Pramod Khosla

**Affiliations:** 1 Department of Nutrition and Food Science, Wayne State University, Detroit, MI 48202, USA; fq8257@wayne.edu; 2 Dietetics Program, Faculty of Health and Medical Sciences, Universiti Kebangsaan Malaysia, Kuala Lumpur 5300, Malaysia; sham_0901@yahoo.com; 3 School of Biosciences, Faculty of Health Sciences, Taylor’s University, Subang Jaya 47500, Malaysia; tilly_karu@yahoo.co.uk

**Keywords:** egg consumption, egg intake, chronic kidney disease, CKD, end-stage renal disease

## Abstract

Patients with chronic kidney disease (CKD) are often instructed to adhere to a renal-specific diet depending on the severity and stage of their kidney disease. The prescribed diet may limit certain nutrients, such as phosphorus and potassium, or encourage the consumption of others, such as high biological value (HBV) proteins. Eggs are an inexpensive, easily available and high-quality source of protein, as well as a rich source of leucine, an essential amino acid that plays a role in muscle protein synthesis. However, egg yolk is a concentrated source of both phosphorus and the trimethylamine N-oxide precursor, choline, both of which may have potentially harmful effects in CKD. The yolk is also an abundant source of cholesterol which has been extensively studied for its effects on lipoprotein cholesterol and the risk of cardiovascular disease. Efforts to reduce dietary cholesterol to manage dyslipidemia in dialysis patients (already following a renal diet) have not been shown to offer additional benefit. There is a paucity of data regarding the impact of egg consumption on lipid profiles of CKD patients. Additionally, egg consumption has not been associated with the risk of developing CKD based on epidemiological studies. The egg yolk also contains bioactive compounds, including lutein, zeaxanthin, and vitamin D, which may confer health benefits in CKD patients. Here we review research on egg intake and CKD, discuss both potential contraindications and favorable effects of egg consumption, and describe the need for further research examining egg intake and outcomes in the CKD and end-stage renal disease population.

## 1. Introduction

Chronic kidney disease (CKD) affects almost 10% of the world’s population [1] and was the sixteenth leading cause of death globally in 2017 [2]. In the United States, over 30 million adults are affected with CKD, which accounts for more than $114 billion in total Medicare spending [3]. In patients with CKD, a low protein diet of 0.6–0.8 g/kg body weight is recommended to delay the progression of kidney failure in non-diabetic CKD patients [4]. However, in end-stage renal disease (ESRD) patients, a higher protein requirement (1.2 g/kg body weight) is necessary to ensure neutral or positive nitrogen balance. For these patients, high biological value (HBV) protein from animals is preferred over the low biological value proteins sourced from plants, as HBV provides essential amino acids. It is recommended that 50% of dietary protein intake should be of HBV proteins [5].

The whole egg is an inexpensive, easily available, versatile and high-quality source of protein. According to the *World Agricultural Supply and Demand Estimates* report, per capita consumption in the United States has steadily increased from ~248 eggs per person, per year in 2008 to ~276 in 2017. Consumption is projected to reach ~281 eggs per person in 2019 [6], likely due to several factors, including the recent popularity of high protein diets. Eggs are also known to have high digestibility value (97%) compared to milk/cheese (95%) or meat/fish (94%) [7] and are a rich source of the essential amino acid leucine, which plays an important role in muscle protein synthesis [8]. Aside from the United States, there are many low- to middle-income countries whose populations depend on eggs as a good source of affordable protein.

However, eggs are also a major source of dietary cholesterol, especially in more developed countries. For almost seventy years, the role of saturated fat and dietary cholesterol, and associations with cardiovascular disease (CVD) risk have been the subject of intense study, debate, controversy and health policy decisions. In the United States, the overall prevalence of CVD was double among those with CKD compared to those without (65.1% versus 32.6%) in 2016 [9]. For those patients receiving hemodialysis (HD), peritoneal dialysis, or a kidney transplant, the prevalence of CVD comorbidities was 70.6%, 57.7%, and 41.4%, respectively [10]. In addition to CVD, diabetes as a traditional risk factor is a well-known etiological cause of CKD across many countries [11], while uremia as a non-traditional cause adds to the morbidity burden of CVD [12]. Thus, additional comorbidities common to CKD such as: anemia [13]; malnutrition [14]; mineral and bone disorders [15]; dyslipidemia [16]; calcium, phosphorus and parathyroid hormone disorders [17]; homocysteine disorders [18]; and inflammation [19], also contribute to the higher rates of CVD in the ESRD population [20].

The 2003 Kidney Disease Outcomes Quality Initiative (K/DOQI) Clinical Practice Guidelines for Managing Dyslipidemias in CKD, which is in current use, was based on the Adult Treatment Panel III (ATP III) Guidelines from the National Cholesterol Education Program [21]. The ATP III guidelines at that time advised initiating therapeutic lifestyle changes, which included limiting saturated fat to less than 7% of calories and cholesterol to less than 200 mg/day if low density lipoprotein cholesterol (LDL-C) was above the goal [22]. However, the 2005 K/DOQI Clinical Practice Guidelines for Cardiovascular Disease in Dialysis Patients declared that due to a lack of evidence regarding CVD and diet in the dialysis population, caution should be taken in extrapolating nutrition guidelines from the general population [23]. More recently, the 2013 Kidney Disease Improving Global Outcomes (KDIGO) guidelines emphasized pharmacological therapy for managing dyslipidemia but did not address dietary intervention strategies [24]. However, ESRD patients with dyslipidemia are currently still advised to limit dietary cholesterol, namely eggs, based on the last ATP III guidelines.

Additionally, the role of dietary cholesterol per se in CVD risk has undergone an evolution in perception. A review of the role of dietary cholesterol in relation to CVD is beyond the scope of this article; however, several excellent articles have been published recently including a Special Issue in this journal [25,26]. Suffice to say, current data suggest that dietary cholesterol, in addition to increasing LDL-C, also raises high density lipoprotein cholesterol (HDL-C), increases the less atherogenic large buoyant LDL, may improve HDL functionality, and minimally affects the ratio of LDL-C/HDL-C. Consistent with these changes in lipoproteins, recent epidemiological studies fail to show any significant impact on CVD risk, with the possible exception of individuals with diabetes [25,27].

Given that eggs are a major source of HBV proteins and their cholesterol content may no longer be a concern in relation to CVD risk, we have evaluated their role in the CKD population and discuss potential benefits, limitations and adverse effects of the consumption of eggs by ESRD patients, as well as provided suggestions for further research. In the following sections, we briefly review the current literature (including the studies described in Table 1) on egg consumption in the non-CKD population and how egg consumption might impact the CKD population in respect to lipoprotein metabolism. We then provide an overview of the nutrients and bioactive egg compounds in eggs and their potential contribution to health outcomes in individuals with CKD. Finally, we review potential associations between egg consumption and the risk of developing CKD.

## 2. Egg Intake and Lipid Metabolism

### 2.1. Non-CKD Population

One large raw egg contains 1.6 grams of saturated fat and 186 mg of cholesterol [35]. Due to the saturated fat content (about 3 g/100 g) and cholesterol content (about 200–300 mg/100 g) of eggs, their consumption in relation to CVD risk remains controversial, despite several meta-analyses suggesting that higher egg consumption of up to one egg per day is not associated with increased CVD risk in the general population [36,37].

For individuals with diabetes, the impact of egg intake on CVD risk is inconclusive. Researchers from the DIABEGG study [38] found that individuals with Type 2 DM (Diabetes Mellitus) had no differences in total cholesterol (TC), LDL-C, triglycerides, or apolipoprotein B when consuming either a high-egg (>12 eggs/week) or low-egg (<2 eggs/week) diet for three months. Dietary cholesterol minimally elevates total cholesterol (TC) levels for most of the population (2/3) in that serum cholesterol is maintained following a higher dietary cholesterol intake via compensatory mechanisms of reduced cholesterol biosynthesis, absorption, and excretion [39,40]. Consumption of up to three eggs per day in both healthy and overweight individuals has been shown to minimally impact the LDL-C to HDL-C ratio [41,42]. Several studies have found that whole egg intake in healthy individuals is associated with favorable effects on lipoprotein particle profiles and HDL functionality, specifically increasing both large LDL and HDL particle size, LCAT (lecithin cholesterol acyltransferase) activity, and plasma apolipoproteins (Apo) AI and AII [43,44]. Two recent, 2018, reviews examining egg consumption, serum lipids, cholesterol homeostasis and heart disease concluded that while egg consumption is not a risk factor for CVD in healthy people, certain groups, such as those with diabetes or hyper-responders to dietary cholesterol, may need to exercise caution. While some of the components of eggs may adversely affect CVD risk, other bioactive egg compounds may have potentially favorable effects [25,45].

### 2.2. CKD Population

Characteristic lipid abnormalities observed in HD patients include low HDL-C, normal or low TC and low LDL-C [16]. Besides decreased HDL-C levels, the HDL is dysfunctional in its anti-oxidative and anti-inflammatory roles [46]. Alterations in lipoprotein metabolism in ESRD include a decrease in Apo AI and AII levels, decreased lecithin–cholesterol acyltransferase (LCAT) and increased cholesteryl ester transfer protein (CETP) activity, downregulation of lipoprotein lipase and the LDL receptor, increased small dense LDL and decreased larger LDL particles [16,47]. Dyslipidemia, common among patients with ESRD, is considered a contributor to the higher rates of CVD observed in this population [20]. The current 2003 K/DOQI dietary guidelines for the management of dyslipidemia in adult CKD patients suggest initiating therapeutic life-style changes (TLC) for: (1) TG > 500 mg/dL, (2) LDL-C > 100 mg/dL, and (3) TG ≥ 200 mg/dL and non-HDL-C ≥ 130 mg/dL. TLC include limiting dietary cholesterol to <200 mg per day [21] which equates to 2–6 whole eggs per week; however, little benefit has been demonstrated in implementing these established lipid lowering recommendations superimposed on the standard renal diet [48]. Increasing dietary cholesterol to 900 mg/day by giving three eggs daily to CKD patients over a four-week period has not been shown to negatively affect serum triglycerides, cholesterol or HDL-C [28]. Additionally, it has been demonstrated in rodent models, (5/6 nephrectomy and nephrotic syndrome) that feedback inhibition of cholesterol biosynthesis is preserved [49,50]. There remains a paucity of research examining the effect of egg consumption on lipoprotein metabolism for the ESRD population, and the potential impact of egg consumption on lipoprotein particle profiles and HDL functionality is unknown.

## 3. Egg Components: Bioactive Molecules and Nutrients

### 3.1. Phospholipids

Egg yolks are one of the richest dietary sources of phospholipids [51] and the glycerophospholipid phosphatidylcholine (PC) is the predominate phospholipid class found in eggs [52]. It is understood from normal population studies that dietary phospholipids (PL) are preferentially incorporated into the HDL fraction of plasma over red blood cells, apoB-containing lipoproteins or total blood [53] and thus raise HDL-C. A reduced HDL phospholipid content and cholesterol efflux capacity have been associated with the development of coronary artery disease for individuals with high HDL-C [54]. Further, daily egg consumption in subjects with Metabolic Syndrome has been shown to promote favorable shifts in HDL lipid composition and PL class distribution, resulting in increased ex-vivo macrophage cholesterol efflux capacity of serum from lipid-loaded macrophages [55].

### 3.2. Trimethylamine N-Oxide (TMAO)

Higher plasma levels of gut-derived uremic toxins from nutrient precursors, namely PC (mainly from egg yolk) and carnitine (mainly from red meat) have been associated with moderate renal impairment [34]. TMAO has been implicated in the progression of renal disease and increased mortality risk in CKD [56]. Because TMAO is a metabolite of PC, which is abundant in egg yolks, an atherogenic link has been proposed between dietary intakes of PC derived choline and CVD [57]. However, some skepticism surrounding TMAO exists, as free TMAO in seafood, considered cardioprotective, is significantly greater than that produced by the gut microbiota from choline and carnitine in red meat and eggs [58]. Despite an increase in plasma trimethylamine and TMAO levels, decreased levels of vascular injury and oxidative stress markers such as sICAM-1 (soluble intercellular adhesion molecule-1), sVCAM-1 (soluble vascular cell adhesion molecule-1), and MDA (soluble vascular cell adhesion molecule-1) levels were observed following a study of oral L-carnitine supplementation provided to HD patients with carnitine deficiency [59]. Current evidence on the role TMAO plays in disease development and progression is inconclusive; therefore, recommendations to restrict TMAO-generating nutrients are currently not suggested [60].

### 3.3. Lutein and Zeaxanthin

Renal failure patients are exposed to increased levels of oxidative stress due to a dysfunctional antioxidant defense system as well as increased production and decreased renal clearance of reactive oxygen species [61]. Plasma lipoproteins are susceptible to oxidative modification, which increases their atherogenicity. Several liposoluble antioxidants, including carotenoids, tocopherols, ubiquinol, and flavonoids, work together to prevent lipid peroxidation [47]. Carotenoids are subdivided into two groups: highly hydrophobic carotenoids, such as β-carotene and lycopene, which are localized in the inner part of LDL, and oxygen-containing xanthophylls, such as lutein and zeaxanthin, which are localized in the outer surface area of both LDL and HDL in an equal distribution [62]. Egg yolks are a rich source of lutein and zeaxanthin with greater bioavailability than from both vegetable sources and supplements, most likely due to the fat content of the egg [63,64,65]. Consumption of one egg daily over five weeks has been shown to increase serum zeaxanthin levels by 38% and lutein levels by 26% [66]. Lutein supplementation has been found to decrease lipid peroxidation and C-reactive protein in healthy non-smokers [67]. Patients with CKD are at higher risk for developing several eye diseases, such as diabetic retinopathy, glaucoma, and age-related macular degeneration (AMD) [68]. Because lutein and zeaxanthin accumulate in the retina, studies have found that these carotenoids may slow the progression of AMD, the leading cause of blindness in older adults [69]. Dietary intake of lutein by HD patients has been shown to be significantly less than controls, potentially due to prescribed restrictions in potassium which may lead to a reduced fruit and vegetable intake [70]. Further research examining the potential of whole egg consumption by the ESRD population to improve intakes of lutein and zeaxanthin, antioxidants which can provide substantial health benefits, is needed.

### 3.4. Vitamin D

Vitamin D deficiency and insufficiency are common among the HD population due to impaired synthesis and metabolism, limited exposure to sunlight, and suboptimal intakes [71]. Current KDIGO guidelines offer no specific vitamin D supplementation recommendations for ESRD patients. Few foods provide vitamin D; however, a large egg (primarily from the yolk) does provide a modest 41 IU towards the Recommended Dietary Allowance requirements of 600–800 IU [35]. Nephropathy is a consequence of vitamin D deficiency which is commonly reported in the diabetic population [72]. A whole egg-containing diet was demonstrated to be more effective than supplemental cholecalciferol at maintaining vitamin D status in a Zucker T2DM rat model [73] and was shown to offer a nephroprotective effect in a T1DM Sprague—Dawley rat model [74].

### 3.5. Protein

According to the Protein Digestibility Corrected Amino Acid Score (PDCAAS), a measure of a protein’s ability to provide adequate levels of essential amino acids, whole egg scores a “1” on a scale of 0 to 1 [75]. As measurement of dietary protein utilization, the biological value of eggs (100) is exceeded only by whey protein (104) [76]. Eggs are also known to have a high digestibility value (97%) compared to milk/cheese (95%) or meat/fish (94%) [7]. ESRD patients, have a higher protein requirement (1.2 g/kg body weight) due to a hypermetabolic state and loss of nutrients during dialysis [4,5]. Egg intake has been demonstrated to have an inverse relationship with dialysis vintage, which may be attributed to loss of interest in food or cooking [77].

Eggs are a rich source of leucine, an essential amino acid that plays an important role in muscle protein synthesis [8]. In healthy young men, whole egg was found to offer greater muscle protein response after resistance exercise compared to egg white, not as a result of leucine availability, but attributed to other bioactive compounds contained in the yolk [78]. Both increased muscle proteolysis [79] and impaired muscle protein synthesis are commonly associated with CKD [80]. Potential favorable effects that egg consumption may confer on preserving CKD patients’ muscle mass and strength have not yet been studied.

## 4. Phosphorus and Phosphorus-to-Protein Ratio

Hyperphosphatemia, a common complication in CKD, is associated with increased risk for all-cause and cardiovascular mortality and fracture-related hospitalization [81]. Despite strategies aimed at reducing serum phosphorus levels, such as use of phosphate binders and removal by dialysis, achieving target phosphate levels is difficult [82]. Additional strategies to control serum phosphorus include reducing dietary phosphorus, a tactic often in conflict with the recommendation to increase protein intake. By choosing foods with a favorable phosphorus-to-protein ratio of less than 10 mg/g, ESRD patients could limit phosphorus intake while consuming adequate protein [29]. Because most of the phosphorus is contained in the yolk (586 mg/100 g), the phosphorus-to-protein ratio content of a whole egg is 13.4 mg/g compared to a more desirable ratio of 1.4 mg/g for an egg white [83]. Successful efforts have been made to prepare low phosphorus egg yolk proteins to be used in marketed egg products which provide a favorable phosphorus-to-protein ratio [33]. One small pilot study of 13 MHD (maintenance hemodialysis) patients found a significant decrease in serum phosphorus after substituting eight ounces of pasteurized liquid egg whites for meat at one meal per day over a six-week period; however, researchers acknowledged the absence of a control, lack of randomization and short duration of this study as shortcomings in drawing conclusions about employing this dietary intervention for long term phosphorus control [30]. An important point to consider is that egg whites are both a low phosphorus food and are free from phosphorus additives. In this regard, the phosphorus bioavailability of foods differs depending upon the source. Organic plant- and animal-based phosphorus have an absorption rate of 40–60%, whereas inorganic phosphorus or food additive-based phosphorus could have an absorption rate of up to 90% [83]. As a strategy to reduce dietary phosphorus intake, several randomized trials have found that replacing foods high in phosphate additives with additive-free foods resulted in significant reductions in serum phosphate concentrations [84] without impacting nutritional status [85]. Approaches aimed at examining associations between diet and ESRD have begun to shift the focus from assessment of individual nutrients to diet patterns and type or quality of foods consumed [86]. As current evidence for traditional dietary restrictions has come into question, liberalization of the renal diet has been suggested [87]. Future studies are needed to determine if a wholefoods, patient-centered and personalized based dietary approach with an emphasis on unprocessed high-quality foods, such as tofu, beans, nuts and eggs could favorably impact renal-specific outcomes [88,89].

## 5. Nutrient Recommendations in CKD

Depending on the stage of CKD, certain nutrients may require dietary restriction, such as sodium and potassium. Table 2 outlines the potential dietary contribution of various nutrients in eggs in relation to renal dietary guidelines as per the National Kidney Foundation. For CKD patients, the amount of whole eggs or egg whites allowed, or suggested, would be largely dependent on the individual nutritional status of the patient. Whole eggs contain calcium, potassium and phosphorus, nutrients which are often restricted in renal diets based on serum levels of potassium, calcium, phosphorus and parathyroid hormone. Most of the protein is found in the egg white, with 20% of calories from protein carried in the yolk versus 84% in the white [35]. Depending on the individualized protein needs of a CKD patient, the choice of whole egg or egg whites provides versality in adding or limiting protein in the renal diet plan. Whole eggs provide a nutrient-dense source of calories to meet the increased energy demands of CKD patients. Additionally, eggs are naturally low in sodium, a nutrient which is restricted in renal diets. Depending on phosphorus restriction and the presence of dyslipidemia, either the whole egg or egg white could be utilized. In comparison to the white, the yolk carries fat soluble vitamins, iron, and zinc, contributing to the Dietary Reference Intakes requirements [5].

## 6. Egg Supplementation Studies

CKD patients undergoing dialysis have higher protein requirements, as they experience protein losses during dialysis. However, inadequate protein intake is an issue in this population as patients experience poor appetite, taste aversion towards protein food sources and limited oral intake due to dietary restrictions to control potassium or phosphorus [29,91]. Therefore, oral protein supplementation (OPS) has been used as a solution to overcome dietary protein inadequacy. To date, only one OPS study using egg albumin as a renal-specific product has been conducted. Jeloka et al. (2013) supplemented HD and peritoneal dialysis patients with either whey protein (46 g protein and 230 kilocalorie) or egg albumin (70 g protein and 316 kilocalorie) for six months and reported no significant difference between the groups, attributed to high non-compliance reported in both groups due to adverse events such as bloating, nausea, or vomiting [31].

## 7. Egg Intake and Risk of Developing CKD

### 7.1. Potential Renal Acid Load (PRAL)

An alkaline diet rich in fruits and vegetables provides a low net acid load, which has been purported to have favorable metabolic effects in patients with CKD. One indicator of dietary acid-base load is PRAL, calculated based on only four nutrients using the following equation: PRAL (mEq/day) = 0.4888 × dietary protein (g/day) + 0.0366 × dietary phosphorus (mg/day) − 0.0205 × dietary potassium (mg/day) − 0.0125 × calcium (mg/day) − 0.0263 × magnesium (mg/day) [92]. In a cohort study of community-dwelling elderly Koreans, researchers evaluating the association between dietary acid load and CKD found that high estimated dietary net acid production, which was correlated with potassium intake but not protein intake, was associated with CKD risk [93]. The Atherosclerosis Risk in Communities (ARIC) study, a community-based observational study of middle-aged adults in the United States, found dietary acid load was associated with incident CKD [94]. Among 12,293 United States adult participants aged >20 years in the National Health and Nutrition Examination Survey 1999–2004, a greater dietary acid load, quantified by estimated net acid excretion, was associated with albuminuria. It is worth noting that no significant associations were found between protein intake and phosphorus intake and albuminuria, indicating that balancing protein intake with base-inducing fruits and vegetables may be important for reducing renal damage [95]. Whole eggs have a relatively neutral pH. Egg whites are one of the few food products that are naturally alkaline and this increases as the egg ages, reaching a pH of up to 9.2. The pH of a fresh egg yolk is about 6.0 and can increase to 6.9 during storage [96]. Given these alkaline properties, one large whole egg makes a relatively low contribution to PRAL (Table 3).

### 7.2. Dietary Quality

Several prospective cohort studies investigating dietary protein sources and risk for incident CKD have failed to find an association between egg intake and CKD risk [32,98]. Using data from the Singapore Chinese Health Study to complete a substitution analysis, Lew et al. estimated that the risk for developing ESRD can be reduced by substituting one serving of red meat with either one serving of poultry (62%), fish (49%), soy and/or legumes (50%) or eggs (45%) [32]. Some insight into future research in patients may be drawn from an animal model study, where Fadupin et al. (2008) showed egg white and fish protein were superior to other protein sources in providing beneficial effects on food intake, anthropometric parameters, blood urea and creatinine levels in nephrectomized rats [99].

## 8. Summary

Dietary intervention trials demonstrating negative effects of egg consumption on CVD risk for individuals with CKD are not available. The current K/DOQI Clinical Practice Guidelines for Managing Dyslipidemias in *CKD* recommend initiating TLC which include limiting saturated fat to less than 7% of calories and cholesterol to less than 200 mg/day if LDL-C, TG, and non-HDL-C levels are above goal. Superimposing these dietary restrictions for ESRD patients has demonstrated little benefit in further lipid lowering beyond pharmacological therapy. Bioactive components of eggs have been shown to demonstrate favorable effects on lipoprotein particle profiles and HDL functionality in heathy adults. Whether these egg compounds might produce similar results in the ESRD population is unknown. As an inexpensive, high quality source of protein, eggs can help meet the increased protein demands of ESRD patients. While the egg yolk does contain higher levels of phosphorus, a nutrient of concern, and choline, a precursor of TMAO, the yolk is also a rich source of bioactive compounds, including lutein, zeaxanthin, and vitamin D, and may help promote muscle protein synthesis. Studies examining dietary patterns and quality have not found an association between egg intake and CKD risk progression. Further research examining egg intake and outcomes in the ESRD population are needed.

## Figures and Tables

**Table 1:** Articles of interest discussed in current review.

Reference	Author, Year	Journal	Study Design	Conclusion
1	Green EM, et al., 1985 [28]	Journal of the American Dietetic Association	Crossover open design (*n* = 4 MHD; *n* = 2 CKD) three eggs daily for 4 weeks (900 mg cholesterol/day)	Ingestion of a high cholesterol diet for 4 weeks was not associated with an increase in serum cholesterol
2	Noori N, et al., 2010 [29]	Iranian Journal of Kidney Diseases	Review article	Egg white has one of the lowest phosphorus-protein ratios
3	Taylor LM, et al., 2011 [30]	Journal of Renal Care	Pilot study: (*n* = 13 MHD) Eight ounces (225 g) of pasteurized liquid egg whites one meal per day for six weeks	Serum phosphorus level decreased significantly by 0.9 mg/dL
4	Jeloka TK, et al., 2013 [31]	Indian Journal of Nephrology	RCT (*n* = 50 HD and PD) supplemented with either whey protein or egg albumin for 6 months based on protein deficits	No increase in total protein or caloric intake in either group because of poor tolerance and severe side effects
5	Lew QJ, et al. 2017 [32]	Journal of the American Society of Nephrology	Epidemiological Study: Singapore Chinese Health Study	Intake of poultry, fish, eggs, or dairy products was not associated with risk of ESRD
6	Long J, et al., 2018 [33]	Journal of the science of food and agriculture	Food science study	Novel method was developed for low-phosphorus yolk protein (LPYP) using alkaline protease auxiliary dephosphorization
7	Pignanelli M, et al. 2018 [34]	Journal of Renal Nutrition	Cohort Study	Moderate impairment of renal function was associated with higher plasma levels of gut-derived uremic toxins. Intake of choline/pre-TMA, largely from egg yolk, contributed significantly to plasma levels of TMAO, hippuric acid, and p-cresyl gluronide

Following a search in PubMed and EMBASE, in October 2018, using various terms (‘egg consumption’, “egg intake’ or ‘egg’ AND ‘CKD’ or ‘chronic kidney disease’ ‘kidney disease’, ‘dialysis’, ‘kidney failure’, ‘renal failure’, ‘end stage renal disease’, ‘hemodialysis’, ‘haemodialysis’, ‘peritoneal dialysis’, ‘pediatric kidney disease’, ‘paediatric kidney disease’), 2440 articles were retrieved. 67 were further evaluated (Supplemental Table S1) following exclusion of articles related to: (i) AKI (acute kidney injury) and transplant patients; (ii) abstracts, case reports, conference proceedings, and letters; (iii) eggs used for the sole purpose of controlling diet composition; (iv) terms using “chicken and egg” and EGG (electrogastrography). Table 1 summarizes the remaining seven pertinent studies. (Egg consumption in the non-CKD population was not evaluated given the vast literature on this subject). MHD = Maintenance; RCT = randomized control trial; PD = peritoneal dialysis.

**Table 2:** Nutrients in one egg as compared to nutrient recommendations for adults with CKD.

Nutrient Recommendations for Adults with CKD [90]	CKD (Stage 4)	HD	Nutrients in One Hard Cooked Egg (1 large/50 g) [35]		Benefit (+) or Concern (−)
Energy kcal/kg ^1^	23–35	30–35 ≥ 60 years 35 < 60 years	Kcals	78	**++**
Protein g/kg ^1,2^	0.8; avoid high protein intake >1.3	1.2 stable 1.2--1.3 acutely ill or PEW	Protein (g)	6.29	**+++**
Sodium mg/day	<2000	750–2000	Sodium (mg)	62	**++**
Iron mg/day	NA	Individualized	Iron (mg)	0.59	**+**
Magnesium mg/day	NA	200–300	Magnesium (mg)	5	**+**
Phosphorus mg/day	800–1000 Maintain blood P/PTH WNL	10–17 mg/kg/day (e.g., 700–1190 g for a 70 kg adult)	Phosphorus (mg)	86	**−−−**
Potassium mg/day	Unrestricted unless serum level is high	Up to 1100 to 1250 mg/day; adjust to serum levels	Potassium (mg)	63	**−**
Calcium mg/day	DRI; maintain serum calcium WNL	≤1000	Calcium (mg)	25	**−**
Zinc mg/day	NA	15	Zinc (mg)	0.53	**+**
2003 Kidney Disease Outcomes Quality Initiative (K/DOQI) Clinical Practice Guidelines for Managing Dyslipidemias in CKD [21]					
Criteria: (1) TG > 500 mg/dL (2) LDL > 100 mg/dL, and (3) TG ≥ 200 mg/dL and non-HDL ≥ 130 mg/dL					
Cholesterol mg/day	200	200	Cholesterol (mg)	186	**−−−**
Saturated Fat	< 7% of calories (e.g., 14 g for an 1800 calorie diet)	< 7% of calories (e.g., 14 g for an 1800 calorie diet)	Saturated Fat (g)	1.63	**−**

^1^ Use standard or adjusted body weight to determine protein and calorie levels. ^2^ All protein recommendations suggest at least 50% high biological value (HBV). PEW = protein energy wasting; PTH = parathyroid hormone; PTH = parathyroid hormone; WNL = within normal limits; DRI = Dietary Reference Intakes.

**Table 3:** The potential renal acid load (PRAL) of food (per average portion size) [97].

Food Type	Average Portion Size	Energy (kcals)	Protein (g)	Potassium (mg)	Phosphorus (mg)	PRAL mEq per Average Portion Size
Beef, lean only	3 oz (85 g)	105	17.3	298	153	6.7
Chicken, meat only	3 oz (85 g)	103	17.4	272	170	7.5
Frankfurters	1 link (85 g)	208	7.22	98	130	5.1
Pork, lean only	3 oz (85 g)	125	17.6	315	170	6.7
Eggs, chicken, whole	1 large (50 g)	73.5	6.25	65	100	2
Eggs, white	1 large (33 g)	12	3	49.5	11	0.4
Eggs, yolk	1 large (17 g)	58	2.7	20.4	85	4
Lentils, green and brown, dried	1/2 cup cooked (32 g dried)	95	7.8	301	112	1.1

## Supplementary Materials

The following are available online at http://www.mdpi.com/2072-6643/10/12/1945/s1, Table S1: Articles assessed for the current review.
