# Supplementary material for: Egg Intake in Chronic Kidney Disease
Source: Nutrients. 2018 Dec 7;10(12):1945. doi: 10.3390/nu10121945 (PMC6315879; doi:10.3390/nu10121945)
Supplement: Supplementary file 1 [file nutrients-10-01945-s001.pdf]

Table S1. Articles assessed for the current review.

	Pub Med & Embase Search Articles screened	Reason for Inclusion	Reason for Exclusion
S1.	Berlyne GM (1969) Nutrition in renal failure. *NutrRev* **27**, 7-12.		Review: decreasing pro to delay progression of CKD
S2.	Green EM, Perez GO, Hsia SL *et al.* (1985) Effect of egg supplements on serum lipids in uremic patients. *Journal of the American Dietetic Association* **85**, 355-357.	Included	
S3.	Giordano C, De Santo NG, Pluvio M (1978) Nitrogen balance in uremic patients on different amino acid and keto acid formulations--a proposed reference pattern. *Am J Clin Nutr* **31**, 1797-1801.		Used "mostly" egg derived protein; N balance study
S4.	Korten G (1988) [Nutritional problems in patients treated with chronic dialysis]. *Zeitschrift fur die gesamte innere Medizin und ihre Grenzgebiete* **43**, 183-187.		Cannot determine relationship with egg alone
S5.	Koschinsky T, He CJ, Mitsuhashi T *et al.* (1997) Orally absorbed reactive glycation products (glycotoxins): an environmental risk factor in diabetic nephropathy. *Proc Natl Acad Sci U S A* **94**, 6474-6479.		Used egg white to measure renal clearance kinetics in CKD patients with DM
S6.	Bammens B, Verbeke K, Vanrenterghem Y (2003) Evidence for impaired assimilation of protein in chronic renal failure. *Kidney International* **64**, 2196-2203.		C-leucine–labeled egg white to measure protein assimilation
S7.	Milias GA, Panagiotakos DB, Pitsavos C *et al.* (2006) Prevalence of self-reported hypercholesterolaemia and its relation to dietary habits, in Greek adults; a national nutrition & health survey. *Lipids Health Dis* **5**, 5.		Does not include CKD
S8.	Pitsavos C, Milias GA, Panagiotakos DB *et al.* (2006) Prevalence of self-reported hypertension and its relation to dietary habits, in adults; a nutrition & health survey in Greece. *BMC public health* **6**.		self-reported HTN and egg intake (FFQ); Does not include CKD
S9.	Bernstein AM, Treyzon L, Li Z (2007) Are high-protein, vegetable-based diets safe for kidney function? A review of the literature. *J Am Diet Assoc* **107**, 644-650.		Review paper examining associations between diet and CKD
S10.	Fadupin GT, Keshinro OO, Arije A et al. (2008) The effects of controlled intake of selected protein foods on nephrectomized rats. African Journal Biomedical Research 11, 47-54.		
S11.	Hwang SY, Taylor CG, Zahradka P *et al.* (2008) Dietary soy protein reduces early renal disease progression and alters prostanoid production in obese fa/fa Zucker rats. *J Nutr Biochem* **19**, 255-262.		Used egg white protein for rat feeding study on early renal disease
S12.	Kazama JJ, Kaneko Y (2009) Egg on the table. *Clinical Journal of the American Society of Nephrology* **4**, 14-15.		"Columbus's fault that he damaged the narrow end of an egg to make it stand on a table."

S13.	Burodom A (2010) Renal Response to Egg White Protein Loading in Healthy Young Adults. *J Med Assoc Thai* **93**, 824-829.		Does not include ESRD
S14.	Dial C, Noël LH (2010) Eggs in the kidney. *Kidney International* **77**, 934.		Renal granuloma around the eggs of Schistosoma hematobium
S15.	Noori N, Sims JJ, Kopple JD *et al.* (2010) Organic and inorganic dietary phosphorus and its management in chronic kidney disease. *Iran J Kidney Dis* **4**, 89-100.	Included (Review)	
S16.	Andrews J, Lee S, Talor Z (2011) The effect of high-protein supplements on serum albumin levels of hemodialysis patients. *Hemodialysis International* **15**, 154.		Abstract: Vital Protein RX protein bars, Nepro, and hardboiled eggs offered to HD patient as snacks to improve alb
S17.	Chang WC (2011) Dietary intake and the risk of hyperuricemia, gout and chronic kidney disease in elderly Taiwanese men. *Aging Male* **14**, 195-202.		Examining dietary intake and CKD risk
S18.	Fadupin GT, Egwu OR (2011) Fish and Egg white Diet In Non-Dialyzing In-Patients with Moderate Chronic Renal Failure. *Afr J Biomed Res* **14**, 95-101.		Provided egg white in combination with fish; questionable study: refers to diet as either egg white, and then whole egg
S19.	Marsset-baglieri A, Diouani A, Fromentin G *et al.* (2011) The satiating effect of egg is higher than fresh cheese when given as isocaloric "solid" preloads in humans. *FASEB Journal* **25**.		Abstract: non-CKD patients
S20.	Singh M, Roeser K, Yadav S *et al.* (2011) Can gastrointestinal clinical presentation predict gastroparesis: Results from a tertiary referral center. *Gastroenterology* **140**, S808.		Abstract: non-CKD; used egg beaters for gastric emptying test
S21.	Taylor LM, Kalantar-Zadeh K, Markewich T *et al.* (2011) Dietary egg whites for phosphorus control in maintenance haemodialysis patients: a pilot study. *J Ren Care* **37**, 16-24.	Included	
S22.	Zajc A, Wujastyk L (2011) Patient education. Protein and money.... What do they have in common? *Journal of renal nutrition : the official journal of the Council on Renal Nutrition of the National Kidney Foundation* **21**, e9-10.		Patient education handout: includes eggs as a protein source
S23.	Apicella L, Guida B, Vitale S *et al.* (2012) Short-term effects of an ω-3 (N-3) rich diet on metabolic and inflammatory markers in renal transplant recipients. *Nephrology Dialysis Transplantation* **27**, ii532-ii533.		effects of a n-3 rich diet on some metabolic and inflammatory markers in kidney transplanted patients; n-6 was reduced by limiting eggs, meat,

			whole grains and cereals
S24.	Bhadra S, Desai H, Feder L *et al.* (2012) Egg club initiative did not improve serum albumin levels in esrd patients. *American Journal of Kidney Diseases* **59**, A22.		Abstract: 14 HD Members of the "egg club" take turns bringing in cooked eggs to share with the other members; The patient-organized egg club did not have any effect on the serum albumin levels
S25.	Mekki K, Remaoun M, Belleville J *et al.* (2012) Hemodialysis duration impairs food intake and nutritional parameters in chronic kidney disease patients. *Int Urol Nephrol* **44**, 237-244.		Followed 20 MHD over 9 yrs; intake Eggs decreases over time
S26.	Nakao T, Kanazawa Y, Wada T *et al.* (2012) Nutritional therapy can prevent or postpone dialysis initiation in stage 5 chronic kidney disease patients. *Clinical Nutrition, Supplement* **7**, 278.		Abstract
S27.	Segawa H, Furutani J, Miyamoto KI (2012) Dietary inorganic phosphorus and intestinal peptide absorption. *Kidney Research and Clinical Practice* **31**, A72.		Intestinal peptide transport activity and PepT1 expression levels on nephrectomized rats
S28.	Cupisti A, Gallieni M, Rizzo MA *et al.* (2013) Phosphate control in dialysis. *Int J Nephrol Renovasc Dis* **6**, 193-205.		Review: discuss Phos/Pro ratio of egg vs egg white
S29.	Cupisti A, Kalantar-Zadeh K (2013) Management of natural and added dietary phosphorus burden in kidney disease. *Semin Nephrol* **33**, 180-190.		Manuscript
S30.	Gonzalez-Campoy JM, St.jeor ST, Castorino K *et al.* (2013) Clinical practice guidelines for healthy eating for the prevention and treatment of metabolic and endocrine diseases in adults: Cosponsored by the American association of clinical endocrinologists/the american college of endocrinology and the obesity society: Executive summary. *Endocrine Practice* **19**, 875-887.		Grade A BEL1 (best evidence level 1) Quality food high in pro, minerals, and vitamins but low in SF, chol, and trans should be recommended for overweight or obese elderly patients to provide adequate protein intake without carrying a high risk for CVD
S31.	Jeloka TK, Dharmatti G, Jamdade T *et al.* (2013) Are oral protein supplements helpful in the management of malnutrition in dialysis patients? *Indian J Nephrol* **23**, 1-4.	Included	

S32.	Köse E, Turgutalp K, Kiykim A *et al.* (2013) The association between feeding habits, nutritional parameters and quality of sleep in hemodialysis patients. *Nephrology Dialysis Transplantation* **28**, i497.		Quality of sleep study HD; The patients with the good quality of sleep consumed significantly more meat-offal-cheese-egg ($p<0.01$) and fruit
S33.	Scialla JJ, Anderson CA (2013) Dietary acid load: a novel nutritional target in chronic kidney disease? *Adv Chronic Kidney Dis* **20**, 141-149.		Review: Dietary Acid Load in CKD
S34.	Wu PY, Yang SH, Chen TH *et al.* (2013) Gender difference in dietary pattern between non-dialysis day in weekday and weekend of hemodialysis patients in north Taiwan. *Annals of Nutrition and Metabolism* **63**, 1290.		110 MHD North Taiwan; 3 day record: Women consume less eggs than men
S35.	Lin HC, Peng CH, Chiou JY *et al.* (2014) Physical activity is associated with decreased incidence of chronic kidney disease in type 2 diabetes patients: A retrospective cohort study in Taiwan. *Primary Care Diabetes* **8**, 315-321.		Study on PA and incident CKD; egg part of FFQ
S36.	Mervish N, McGovern KJ, Teitelbaum SL *et al.* (2014) Dietary predictors of urinary environmental biomarkers in young girls, BCERP, 2004-7. *Environmental Research* **133**, 12-19.		measured urinary exposure biomarkers for phthalates, parabens, and BPA and assessed dietary intake using 24-h in girls
S37.	Turney BW, Appleby PN, Reynard JM *et al.* (2014) Diet and risk of kidney stones in the Oxford cohort of the European Prospective Investigation into Cancer and Nutrition (EPIC). *European Journal of Epidemiology* **29**, 363-369.		Diet and risk of kidney stones
S38.	Van Wyk K, Schalinske K (2014) Whole egg protein markedly increases blood vitamin D concentrations in male Sprague-Dawley rats. *FASEB Journal* **28**.		Abstract: Whole egg 4 fold increase in activity of hepatic glycine N-methyltransferase vs egg white
S39.	Jones S, Schalinske K (2015) Whole egg consumption completely prevents vitamin D deficiency in type 2 diabetic rats. *FASEB Journal* **29**.		Abstract: Whole egg resulted in a 2-fold increase in circulating 25D in the lean control group vs casein based diet
S40.	Fouque D, Mitch WE (2015) Low-protein diets in chronic kidney disease: Are we finally reaching a consensus? *Nephrology Dialysis Transplantation* **30**, 6-8.		Makes reference to efforts in the 1940s to design regiments like the egg-potato diet based on meals that were poor in the

			amount of proteins for patients with chronic kidney disease (CKD).
S41.	Moraes C, Fouque D, Amaral AC *et al.* (2015) Trimethylamine N-Oxide From Gut Microbiota in Chronic Kidney Disease Patients: Focus on Diet. *J Ren Nutr* **25**, 459-465.		Review: potential concerns about TMAO in nondialysis CKD patients
S42.	Severins N, Mensink RP, Plat J (2015) Effects of lutein-enriched egg yolk in buttermilk or skimmed milk on serum lipids & lipoproteins of mildly hypercholesterolemic subjects. *Nutrition, Metabolism and Cardiovascular Diseases* **25**, 210-217.		Non CKD population
S43.	D'Alessandro C, Piccoli GB, Calella P et al. (2016) "Dietaly": Practical issues for the nutritional management of CKD patients in Italy. BMC Nephrology 17.		Review of diet for CKD Italy "we recommend an occasional consumption of whole egg as the yolk is rich in phosphorus"
S44.	Gaipov A, Solak Y, Zhampeissov N *et al.* (2016) Renal functional reserve and renal hemodynamics in hypertensive patients. *Ren Fail* **38**, 1391-1397.		Used egg white protein to measure renal function reserve
S45.	Garagarza C, Silva J, Valente A *et al.* (2016) Effect of a protein-rich meal intake during hemodialysis treatment. *Nephrology Dialysis Transplantation* **31**, i299.		Study to evaluate the effect of intradialytic oral nutrition supplementation in HD patients; included an egg sandwich with ONS
S46.	Golzarand M, Bahadoran Z, Mirmiran P *et al.* (2016) Protein Foods Group and 3-Year Incidence of Hypertension: A Prospective Study From Tehran Lipid and Glucose Study. *Journal of renal nutrition : the official journal of the Council on Renal Nutrition of the National Kidney Foundation* **26**, 219-225.		Risk of possible effect of protein foods group and its subgroups on risk of HTN after 3-year follow-up in Iranian adults: consumption of eggs is inversely associated with the risk of high blood pressure
S47.	Grant WB (2016) Using Multicountry Ecological and Observational Studies to Determine Dietary Risk Factors for Alzheimer's Disease. *Journal of the American College of Nutrition* **35**, 476-489.		Review: Non CKD; Dietary Risk Factors and Alzheimer's Disease -- The most important dietary link to AD appears to be meat consumption, with eggs and high-fat dairy

			also contributing
S48.	Saande CJ, Schalinske KL, Rowling MJ (2016) Whole egg consumption attenuates vitamin d insufficiency in type 1 diabetic rats. *FASEB Journal* **30**.		Abstract: T1DM rats and vit D study
S49.	Shivappa N, Hébert JR, Askari F *et al.* (2016) Increased inflammatory potential of diet is associated with increased risk of prostate cancer in Iranian men. *International Journal for Vitamin and Nutrition Research* **86**, 161-168.		Non CKD population
S50.	Smith RE, Rouchotas P, Fritz H (2016) Lecithin (Phosphatidylcholine): Healthy dietary supplement or dangerous toxin? *Natural Products Journal* **6**, 242-249.		Review: "People who have a kidney disease or are highly susceptible to CVD may want to restrict their consumption of red meat and egg yolks, as well as avoid lecithin dietary supplements"
S51.	Spence JD (2016) Recent advances in pathogenesis, assessment, and treatment of atherosclerosis. *F1000Research* **5**.		Review article non CKD population
S52.	Cupisti A, D'Alessandro C, Gesualdo L *et al.* (2017) Non-traditional aspects of renal diets: Focus on fiber, alkali and Vitamin K1 intake. *Nutrients* **9**.		Review: Diets for renal insufficiency
S53.	Fernandes AS, Ramos CI, Nerbass FB *et al.* (2017) Diet Quality of Chronic Kidney Disease Patients and the Impact of Nutritional Counseling. *J Ren Nutr*.		Diet quality on CKD; meat/egg group one of 10 component scores
S54.	Lew QJ, Jafar TH, Koh HW *et al.* (2017) Red Meat Intake and Risk of ESRD. *J Am Soc Nephrol* **28**, 304-312.	Included: Epi study	
S55.	Martins AM, Bello Moreira AS, Canella DS *et al.* (2017) Elderly patients on hemodialysis have worse dietary quality and higher consumption of ultraprocessed food than elderly without chronic kidney disease. *Nutrition* **41**, 73-79.		Investigated the dietary quality and consumption of ultraprocessed food by elderly patients on HD and those without CKD. Elder-HD group showed lower scores of egg intake than non CKD
S56.	Shivappa N, Hébert JR, Rosato V *et al.* (2017) Dietary Inflammatory Index and Renal Cell Carcinoma Risk in an Italian Case–Control Study. *Nutrition and Cancer* **69**, 833-839.		Association between the dietary inflammatory index (DII) and renal cell carcinoma
S57.	Broberg B, Madsen J, Fuglsang S *et al.* (2018) Gastrointestinal motility in patients with end-stage renal disease on chronic hemodialysis. *Nephrology Dialysis Transplantation* **33**, i317-i318.		Used labeled egg omelet to measure gastric motility

S58.	Houston M, Minich D, Sinatra ST *et al.* (2018) Recent Science and Clinical Application of Nutrition to Coronary Heart Disease. *Journal of the American College of Nutrition* **37**, 169-187.		Healthy population & Egg intake: No association with increased CHD risk, except possibly for diabetics
S59.	Jung-Hyun K, Dong-Ryeol R, Hyun-Jung K *et al.* (2018) Development of semi-quantitative food frequency questionnaire for korean hemodialysis patients. *Nephrology Dialysis Transplantation* **33**, i277.		Validation of Korean FFQ in HD
S60.	Kawamura H, Tanaka S, Ota Y *et al.* (2018) Dietary intake of inorganic phosphorus has a stronger influence on vascular-endothelium function than organic phosphorus. *Journal of Clinical Biochemistry and Nutrition* **62**, 167-173.		Used dried egg yolk as organic and inorganic P sources
S61.	Long J, Wang F, Jiao A *et al.* (2018) Preparation, characterization and physicochemical properties of novel low-phosphorus egg yolk protein. *Journal of the science of food and agriculture*.	Included: Novel method was developed for low-phosphorus yolk protein (LPYP) using alkaline protease auxiliary dephosphorization	
S62.	Puchulu MB, Ogonowski N, Sanchez-Meza F *et al.* (2018) Dietary Phosphorus to Protein Ratio for the Mexican Population with Chronic Kidney Disease. *Journal of the American College of Nutrition*.		Outlines phosphorus to protein ratio in foods commonly used by the Mexican population
S63.			Eggs as part of a diet pattern associated with lower serum urate levels Genetic variants explain substantially more of the variation in serum urate levels compared with dietary patterns
S64.	Pignanelli M, Bogiatzi C, Gloor G *et al.* (2018) Moderate Renal Impairment and Toxic Metabolites Produced by the Intestinal Microbiome: Dietary Implications. *J Ren Nutr*.	Included	
S65.	Saande CJ, Jones SK, Rowling MJ *et al.* (2018) Whole Egg Consumption Exerts a Nephroprotective Effect in an Acute Rodent Model of Type 1 Diabetes. *J Agric Food Chem* **66**, 866-870.		
S66.	Shi Z, Taylor AW, Riley M *et al.* (2018) Association between dietary patterns, cadmium intake and chronic kidney disease among adults. *Clinical Nutrition* **37**, 276-284.		Dietary pattern associations and risk of CKD A modern dietary pattern (high intake of fruit, soy

			milk, egg, milk and deep fried products) was inversely associated with CKD
S67.	Spence JD (2018) Egg Consumption and Cardiovascular Risk. *Canadian Journal of Diabetes* **42**, 222.		Letter to the editor
